# State Variability in Emergency Department Visits among Assisted Living Residents With Dementia

**DOI:** 10.1093/geroni/igaa057.2526

**Published:** 2020-12-16

**Authors:** Cassandra Hua, Wenhan Zhang, Portia Cornell, Momotazur Rahman, David Dosa, Kali Thomas

**Affiliations:** 1 Brown University School of Public Health, Providence, Rhode Island, United States; 2 Brown University, Providence, Rhode Island, United States; 3 Providence VA Medical Center, Providence, Rhode Island, United States; 4 Brown University, Barrington, Rhode Island, United States

## Abstract

Emergency department (ED) visits are associated with poor outcomes; however, state variation in ED use among assisted living (AL) residents is not well understood. Using 2017 Medicare data, we identified a cohort of 88,880 beneficiaries with dementia residing in larger ALs (25+ beds) and calculated risk-adjusted rates of all-cause and injury-related ED use per 100 person years, by state, adjusting for demographics and chronic conditions. Risk-adjusted state rates of all-cause ED visits ranged from 129.5 visits/100 person-years (95%CI=114.6,148.2) in New Mexico to 246.1 visits/100 person-years (95%CI= 224.9,274.8) in Rhode Island. The risk-adjusted rate of injury-related ED visits ranged from 91.4 visits/100 person-years (95%CI=83.0,101.4) in New Mexico to 135.9 visits/100 person-years (95%CI=126.9,146.6) in Montana. Potential reasons for these state variations will be discussed. Part of a symposium sponsored by Assisted Living Interest Group.

